# Can stress echocardiography identify patients who will benefit from percutaneous mitral valve repair?

**DOI:** 10.1007/s10554-018-1507-x

**Published:** 2018-11-29

**Authors:** J. F. Velu, J. Baan Jr, H. A. C. M. de Bruin-Bon, M. S. van Mourik, M. Nassif, K. T. Koch, M. M. Vis, R. B. van den Brink, S. M. Boekholdt, J. J. Piek, B. J. Bouma

**Affiliations:** 1 Amsterdam UMC, Heart Center, Department of Cardiology, Amsterdam Cardiovascular Sciences, University of Amsterdam, Amsterdam, The Netherlands; 2 Department of Cardiology, Academic Medical Center, Meibergdreef 9, 1105 AZ Amsterdam, The Netherlands

**Keywords:** Mitral regurgitation, Echocardiography, Transcatheter valve interventions, MitraClip

## Abstract

The aim of the current study was to investigate whether stress echocardiography improves selection of patients who might have clinical benefit from percutaneous mitral valve repair with the MitraClip. In total, 39 patients selected for MitraClip implantation underwent preprocedural low-dose stress (dobutamine or handgrip) echocardiography from which stroke volume, ejection fraction and MR grade were measured. Outcome after MitraClip implantation was determined by New York Heart Association classification and Quality of Life questionnaires. Clinical benefit from MitraClip treatment was defined as survival and NYHA class I–II at 6 months follow-up. In total, 36 patients with a technically successful procedure were included in the analysis (mean age 79 ± 8 years, 47% male, 50% functional MR). Clinical benefit was achieved in 18 patients. All seven patients with MR decreasing during stress remained in NYHA III–IV or died within 6 months, while 62% (18 out of 29) of the patients with stable or increased MR during stress had clinical benefit (p = 0.008). Significant increase in Quality of Life on 4/8 subscales of the RAND Short Form-36 questionnaire was observed: Physical Functioning (p < 0.001), Social Functioning (p < 0.001), Mental Health (p = 0.022) and Vitality (p = 0.026) was seen in patients with an increase in stroke volume during stress echocardiography. Patients with a decreased MR during preprocedural stress echocardiography remained more symptomatic than patients with a stable or increased MR during stress. Stress echocardiography may support patient selection for percutaneous mitral valve repair.

**Electronic supplementary material:**

The online version of this article (10.1007/s10554-018-1507-x) contains supplementary material, which is available to authorized users.

## Introduction

MitraClip is a treatment option for patients with symptomatic, moderate-severe to severe mitral regurgitation (MR) in whom risks of conventional surgery are too high [1–3]. However, not all patients have clinical benefit after a technically successful procedure [4–7]. Improvement of selection of patients who have clinical benefit from the MitraClip is needed as unnecessary harm to the patient should be avoided and resources are limited [5, 6]. The process of patient selection is complex because the majority of patients is frail, and suffer from comorbidities that also contribute to their symptoms, including pulmonary disease, coronary artery disease and end-stage heart failure [8, 9]. In addition, MR itself is often complex because of anatomic heterogeneity, large variation in etiologies (functional and degenerative MR) and varying response to exercise [10–12].

In literature, it was emphasized that the majority of patients experience symptoms during exercise, whereas most echocardiograms are performed in rest [13–16]. Some studies suggest that stress echocardiography might be useful for risk-stratification in patients with MR [13, 14, 16–19]. In these patients with MR, echocardiographic parameters, e.g. increase in systolic pulmonary artery pressure, increase in stroke volume and increase in MR severity during exercise, have been described to identify patients who were at higher risk of cardiovascular death [13, 18, 19]. However, these studies were focused on conservatively treated patients with MR, while there is need for a risk-stratification tool in patients with MR who undergo a MitraClip procedure to improve future selection of patients.

The aim of the current study was to investigate whether stress echocardiography may improve the selection of patients who will have clinical benefit from percutaneous mitral valve repair with the MitraClip.

## Methods

For this prospective study patients who were scheduled for MitraClip treatment between June 2015 and December 2016 were approached. In total, 39 patients gave written informed consent and underwent stress echocardiography prior to the MitraClip implantation. The study complied with the ethical guidelines of the 1975 Declaration of Helsinki regarding investigation in humans and was approved by the Medical Ethics Committee (NL52635.018.15).

Transthoracic low-dose stress echocardiography (Vivid E9; GE Healthcare, Horten, Norway) using handgrip and/or dobutamine were added to the preprocedural evaluation of the patients. Handgrip exercise was performed 3–5 min, depending on patients’ capabilities. Initial dose of intravenous dobutamine was 5 µg/kg/min which was increased in phases of 3 min to 10 and 15 µg/kg/min. Patients were instructed to withhold their beta-blockers prior to the stress echocardiography. Changes in stroke volume (pulse wave Doppler apical 5 chamber view), ejection fraction (Simpson’s rule biplane method) and MR grade were determined. Stroke volume was corrected for heart rate, with 75 beats per minute (bpm) as reference. MR severity was graded as none, mild (1), moderate (2), moderate to severe (3) or severe (4) based on qualitative, semiquantitative and quantitative parameters according to the ESC guidelines [20]. Echocardiographic recordings were digitalized and analyzed offline. All echocardiographic measurements were performed by a single experienced investigator (JFV) and reviewed by an experienced sonographer (HAdB), both blinded for the clinical outcome.

Improvement after MitraClip implantation was assessed by New York Heart Association (NYHA) classification, Quality of Life (QoL) questionnaires, 6 min walk test (6MWT) and VO_2_ max cycling test (VO_2_ max). The QoL evaluation was based on the Minnesota Living with Heart Failure Questionnaire (MLHFQ), RAND Short Form-36 (SF-36) and EuroQol-5D (EQ-5D). The VO_2_ max cycling test started at a voltage of 0 W aiming for a test between 6 and 8 min. At follow-up, the same measurements were performed.

Baseline characteristics of the patients were entered into a dedicated, prospective database. All patients were invited for clinical evaluation and transthoracic echocardiography (TTE) at 1 month, 6 months and 12 months post MitraClip implantation. Patients were followed until either death or end of follow-up (29th of November 2017). Patients in whom implantation was technically successful were included in the analysis. A technically successful implantation was defined as a procedural reduction to MR grade ≤ 2. Clinical benefit from MitraClip treatment was defined as survival and NYHA class I or II at 6 months follow-up.

### Statistical analysis

Continuous variables were expressed as mean ± standard deviation (SD) or as median (25th–75th percentile). Categorical variables were presented as absolute numbers and percentages. The Fisher’s Exact test was used to compare unpaired categorical data. A Student *t* test was used to compare continuous variables if normally distributed and a Mann–Whitney U test if not normally distributed. Correlations were analyzed using linear regression analysis. Differences were considered statistically significant at p values < 0.05. All statistical analyses were performed using SPSS software (IBM SPSS Statistics version 24, New York, USA).

## Results

In total, 39 patients underwent a stress echocardiography, of whom 36 were technically successfully treated and included in the analysis. 47% was male and the mean age was 79 ± 8 years (Table 1). Functional MR was present in 50% of the patients. No procedural mortality occurred and survival after 30 days and 1 year was 94% and 83% respectively. Three of the seven deceased patients died due to a cardiac cause (heart failure). Median follow-up was 551 (354–727) days. At baseline, 78% of the patients were in NYHA class III or IV. The percentage of patients in NYHA class III or IV after 1 month and 12 months was 33% and 34% respectively (Supplementary Fig. 1). MR grade 4 was present in 83% of the patients at baseline, in 15% of the patients at 1 month follow-up and in 29% of the patients at 12 month follow-up (Supplementary Fig. 2).

**Table 1:** Baseline characteristics—data are presented as mean ± standard deviation, median (25th–75th percentile), or number (percentage)

Variable	Patients undergoing successful MitraClip	Clinical benefit	No clinical benefit	p-value
	(n = 36)	(n = 18)	(n = 18)	
Age at procedure (years)	79 ± 8	81 ± 8	77 ± 8	ns
Men	17 (47%)	9 (50%)	8 (44%)	ns
EuroSCORE I	15 ± 12	16 ± 14	15 ± 10	ns
EuroSCORE II	6 ± 5	5 ± 4	7 ± 5	ns
Clinical history				
Atrial fibrillation	24 (67%)	9 (50%)	15 (83%)	ns
Chronic obstructive pulmonary disease	5 (14%)	0 (0%)	5 (28%)	0.045
Coronary artery disease	15 (42%)	5 (28%)	10 (56%)	ns
Diabetes mellitus	7 (19%)	2 (11%)	5 (28%)	ns
Previous coronary artery bypass graft	9 (25%)	3 (17%)	6 (33%)	ns
Previous percutaneous coronary intervention	8 (22%)	3 (17%)	5 (28%)	ns
Previous stroke	5 (14%)	4 (22%)	1 (6%)	ns
Previous valve surgery	1 (3%)	0 (0%)	1 (6%)	ns
New York Heart Association class ≥ III/IV	28 (78%)	12 (66%)	16 (89%)	ns
6MWT (m)	321 ± 130	386 ± 91	255 ± 132	ns
VO_2_ max cycling test (mL/kg/min)	11 ± 3	11 ± 3	10 ± 3	ns
N-terminal pro-B-type natriuretic peptide (ng/L)	2337 (927–6358)	1979 (1175–3869)	3303 (862–7311)	ns
Echocardiographic variables				
MR grade 4	30 (83%)	15 (83%)	15(83%)	ns
Tricuspid regurgitation grade 4	4 (11%)	2 (11%)	2 (11%)	ns
MR etiology				ns
Degenerative	18 (50%)	12 (67%)	6 (33%)	
Functional	18 (50%)	6 (33%)	12 (67%)	
Systolic pulmonary artery pressure (mmHg)	43 ± 14	42 ± 14	43 ± 13	ns
Cardiac output (L/min)	4.3 ± 1.4	4.2 ± 1.4	4.4 ± 1.5	ns
Left ventricular ejection fraction (%)	40 ± 12	42 ± 13	38 ± 11	ns
Vena contracta width (mm)	6.5 ± 1.4	6.3 ± 1.4	6.7 ± 1.5	ns

Data were available in up to 36 patients

*ns* not significant

Low-dose stress echocardiography using handgrip was conducted in 15 patients, low-dose stress echocardiography using dobutamine in 6 patients and low-dose stress echocardiography using both handgrip and dobutamine in 18 patients, driven by their eligibility and consent. Findings during maximum stress were used for the analysis. The maximum stress was defined as the maximal achieved change in ejection fraction of stroke volume. Clinical benefit from MitraClip treatment (survival and NYHA I or II at 6 months follow-up) was achieved in 18 of these 36 patients.

**Fig. 1:**
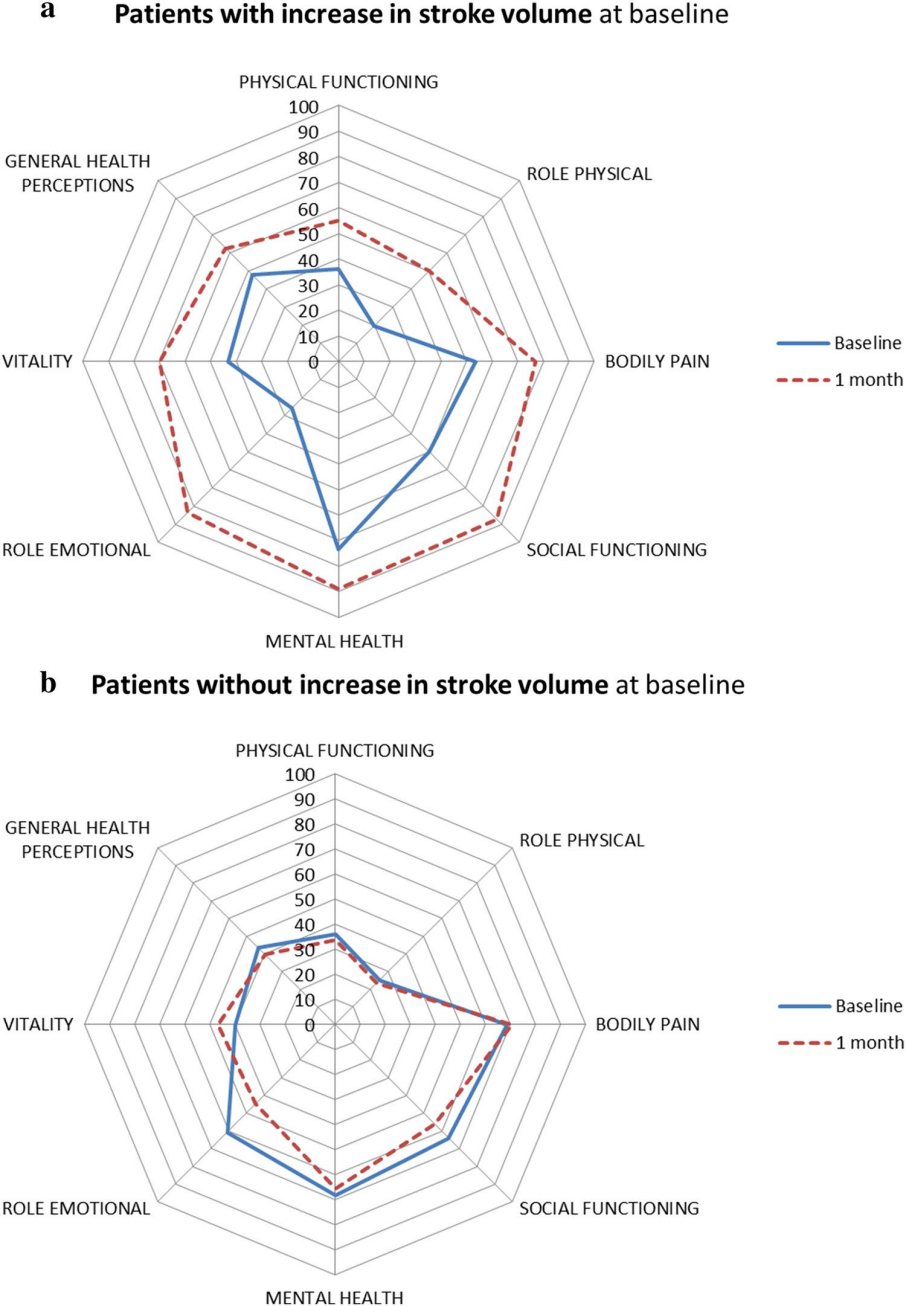
Stroke volume. RAND Short Form-36 Quality of Life questionnaire as assessed at baseline (solid line) and 1 month after the MitraClip implantation (dotted line). The significant increased four subscales were: Physical Functioning (p < 0.001), Social Functioning (p < 0.001), Mental Health (p = 0.022) and Vitality (p = 0.026). **a** Patients with an increase in stroke volume (> 40%) at baseline (n = 10); **b** patients without an increase in stroke volume (≤ 40%) at baseline (n = 25)

A significant increase in Quality of Life on 4/8 subscales of the RAND Short Form-36 questionnaire was observed: Physical Functioning (p < 0.001), Social Functioning (p < 0.001), Mental Health (p = 0.022) and Vitality (p = 0.026) was associated with an increase in stroke volume during stress echocardiography (Fig. 1). In total, 80% of the patients with an increase in stroke volume had a stable or increased MR during stress.

**Fig. 2:**
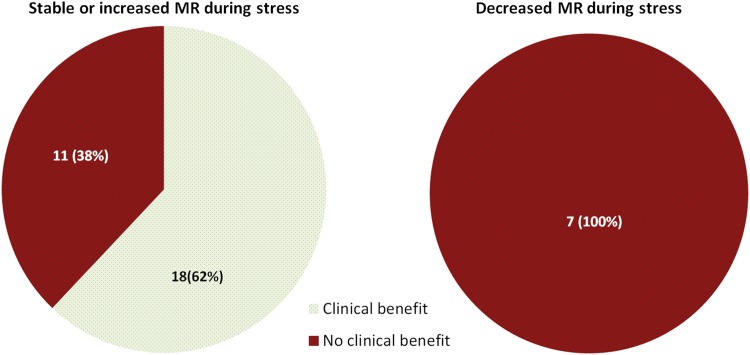
MR grade. Clinical benefit is defined as survival and NYHA I or II at 6 months. Stable or increased MR during stress: clinical benefit in 62% (18/29) versus decreased MR during stress: clinical benefit in 0% (0/7) p = 0.008 (Fisher’s Exact test). *LVEF* left ventricular ejection fraction, *MR* mitral regurgitation

All seven patients with a decreased MR grade during stress [both functional (4/7) and degenerative (3/7) MR] remained in NYHA III or IV or died within 6 months, while 62% (18/29) of the patients with stable or increased MR during stress had clinical benefit (p = 0.008, Fig. 2).

Ejection fraction and change in ejection fraction during stress echocardiography were not associated with clinical benefit from MitraClip treatment. The stress echocardiography parameters were not significantly associated with change in 6MWT, VO_2_ max, MLHFQ, EQ-5D score or NT-proBNP levels.

## Discussion

Our main finding is that a stable or increased MR during stress was associated with clinical benefit, while MR grade decreasing during stress was associated with limited clinical benefit. We also showed that improvement in QoL was associated with increase in stroke volume.

### Stroke volume

Improvement in QoL after MitraClip was associated with increase in stroke volume during stress echocardiography. Patients without increase in stroke volume (≤ 40%) had a higher heart rate in rest (83 ± 15 bpm in rest) and at maximum stress (101 ± 25 bpm) compared to those with an increase of > 40% in stroke volume (in rest 72 ± 10 bpm; 90 ± 14 bpm during stress). Literature is inconsistent regarding the expected increase in stroke volume, which may be limited or even absent when stroke volume reaches a plateau [21]. This might be the physiological explanation for patients without an increase in stroke volume. It could be that they were already in a plateau phase, therefore stroke volume per heartbeat could no longer be increased, despite stress.

Another study showed that an increase in stroke volume after MitraClip implantation (stroke volume at discharge divided by stroke volume at baseline, discharge/baseline ratio) was associated with a more favourable outcome. Further, patients with an increase in stroke volume after the MitraClip implantation had a significantly more severe MR at baseline and a significantly lower stroke volume at baseline [22].

### MR grade

A decreased MR during stress echocardiography prior to MitraClip implantation was associated with limited clinical benefit. One of the mechanisms behind this might be that the mitral annulus size reduced during stress, with an accompanying improvement of coaptation of the leaflets resulting in MR decrease. In these patients, MR might have contributed less to their complains of symptoms during exercise, explaining the lack of benefit from a technically successful MitraClip procedure.

The dynamic character of both degenerative and functional MR was in a previous study suggested as an explanation for symptoms during exercise [23]. The study of Magne et al. focused on conservatively treated patients and found that increased MR during stress was associated with impaired outcome [14]. Other studies had similar findings and determined an effective regurgitation orifice area by ≥ 13 mm^2^ as cut-off value [15, 24]. The dynamic character of MR is also noticeable in the anaesthetic-related damping of periprocedural MR which leads to underestimation of MR reduction the following day [12].

### Ejection fraction

An impaired LVEF in rest echocardiography was not a predictor for survival after MitraClip implantation [5, 6, 25]. The current study showed that increase of the LVEF during stress was also not a predictor for survival after MitraClip. LVEF increase during stress was shown as an important predictor in the field of response to cardiac resynchronization therapy [26, 27]. On top of that, other studies in patients with MR demonstrated that LVEF increase during stress was associated with more vital ventricles and better event-free survival. We could not confirm this, probably due to low patient numbers [28–30]. It is certainly possible that the selection of the best subpopulation to get a MitraClip treatment should be based on several variables. For example, patients with contractile reserve and also a stable of increased MR during stress. This could be a subgroup with vital ventricles and also a clear contribution of the MR to their symptoms.

### Considerations and limitations

Only technically successful implantations (procedural reduction to MR grade ≤ 2) were included in the analysis as a lack of MR reduction precluded clinical benefit. Because of the limited number of patients in this analysis, further differentiation in analyses e.g. types of MR and types of stress during echocardiography was not possible. The study was a single-center study with a limited number of patients who did not all undergo a low-dose stress echocardiography with both handgrip and dobutamine. The fact that not all patients underwent a dobutamine stress echocardiography may have led to confounders.

### Future research

Future research should focus on patients with functional MR because of the dynamic behaviour and prevalence of this etiology. Furthermore, future research should use dobutamine stress echocardiography in all patients to prevent confounders and because of the quantitative and protocoled compared to handgrip stress echocardiography. A larger sample size is necessary to perform multivariate analysis, which is important to determine the independent predictors of clinical benefit. Moreover, a comparison between dobutamine stress echocardiography and stress testing with magnetic resonance imaging can be an interesting topic, especially regarding viability assessment.

## Conclusion

Our main finding was that patients with a decreased MR during preprocedural stress echocardiography remained more symptomatic than patients with a stable or increased MR during stress. Therefore only patients with moderate-severe to severe MR during rest as well as during stress should be selected for MitraClip.

Further, improvement in QoL after MitraClip was associated with increase in stroke volume during stress echocardiography. Hence, stress echocardiography may support patient selection for percutaneous mitral valve repair.

## Electronic supplementary material

Below is the link to the electronic supplementary material.

Supplementary Fig. 1—NYHA: New York Heart Association (TIF 496 KB)

Supplementary Fig. 2—MR: mitral regurgitation (TIF 526 KB)

## References

[1] Feldman T, Kar S, Rinaldi M, Fail P, Hermiller J, Smalling R (2009). Percutaneous mitral repair with the MitraClip system. Safety and midterm durability in the initial EVEREST (Endovascular Valve Edge-to-Edge REpair Study) cohort. J Am Coll Cardiol.

[2] Lim DS, Reynolds MR, Feldman T, Kar S, Herrmann HC, Wang A (2014). Improved functional status and quality of life in prohibitive surgical risk patients with degenerative mitral regurgitation after transcatheter mitral valve repair. J Am Coll Cardiol.

[3] Mauri L, Foster E, Glower DD, Apruzzese P, Massaro JM, Herrmann HC (2013). 4-Year results of a randomized controlled trial of percutaneous repair versus surgery for mitral regurgitation. J Am Coll Cardiol.

[4] Feldman T, Kar S, Elmariah S, Smart SC, Trento A, Siegel RJ (2015). Randomized comparison of percutaneous repair and surgery for mitral regurgitation 5-year results of EVEREST II. J Am Coll Cardiol.

[5] Boerlage-vanDijk K, Wiegerinck EMA, Araki M, Meregalli PG, Bindraban NR, Koch KT (2015). Predictors of outcome in patients undergoing MitraClip implantation: an aid to improve patient selection. Int J Cardiol.

[6] Velu JF, Kortlandt FA, Hendriks T, Schurer RAJ, van Boven AJ, Van den Branden BJL (2017). Percutaneous mitral valve repair. J Am Coll Cardiol.

[7] Rahhab Z, Kortlandt FA, Velu JF, Schurer RAJ, Delgado V, Tonino P (2017). Current mitraclip experience, safety and feasibility in the Netherlands. Netherlands Heart J.

[8] Velu JF, Haas SD, Van Mourik MS, Koch KT, Vis MM, Henriques JP (2017). Elixhauser comorbidity score is the best risk score in predicting survival after MitraClip implantation. Struct Heart.

[9] Neuss M, Schau T, Schoepp M, Seifert M, Hölschermann F, Meyhöfer J (2013). Patient selection criteria and midterm clinical outcome for MitraClip therapy in patients with severe mitral regurgitation and severe congestive heart failure. Eur J Heart Fail.

[10] Lancellotti P, Magne J (2013). Stress echocardiography in regurgitant valve disease. Circ Cardiovasc Imaging.

[11] Lancellotti P, Fattouch K, La Canna G (2015). Therapeutic decision-making for patients with fluctuating mitral regurgitation. Nat Rev Cardiol.

[12] Levine RA, Schwammenthal E (2005). Ischemic mitral regurgitation on the threshold of a solution: from paradoxes to unifying concepts. Circulation.

[13] Picano E, Pibarot P, Lancellotti P, Monin JL, Bonow RO (2009). The emerging role of exercise testing and stress echocardiography in valvular heart disease. J Am Coll Cardiol.

[14] Magne J, Mahjoub H, Dulgheru R, Pibarot P, Pierard LA, Lancellotti P (2014). Left ventricular contractile reserve in asymptomatic primary mitral regurgitation. Eur Heart J.

[15] Lancellotti P, Lebrun F, Piérard LA (2003). Determinants of exercise-induced changes in mitral regurgitation in patients with coronary artery disease and left ventricular dysfunction. J Am Coll Cardiol.

[16] Lancellotti P, Dulgheru R, Go YY, Sugimoto T, Marchetta S, Oury C (2017). Stress echocardiography in patients with native valvular heart disease. Heart.

[17] Geleijnse ML, Fioretti PM, Roelandt JR (1997). Methodology, feasibility, safety and diagnostic accuracy of dobutamine stress echocardiography. J Am Coll Cardiol.

[18] Jansen R, Kracht PAM, Cramer MJ, Tietge WJ, van Herwerden L, Klautz RJM (2013). The role of exercise echocardiography in the management of mitral valve disease. Neth Heart J.

[19] Garbi M, Chambers J, Vannan MA, Lancellotti P (2015). Valve stress echocardiography: a practical guide for referral, procedure, reporting, and clinical implementation of results from the HAVEC group. JACC Cardiovasc Imaging.

[20] Baumgartner H, Falk V, Bax JJ, De Bonis M, Hamm C, Holm PJ (2017). 2017 ESC/EACTS Guidelines for the management of valvular heart disease. Eur Heart J.

[21] Vieira SS, Lemes B, de TC de Carvalho P, de Lima RN, Bocalini DS, Junior JAS (2016). Does stroke volume increase during an incremental exercise? A systematic review. Open Cardiovasc Med J.

[22] Kubo S, Nakamura M, Shiota T, Itabashi Y, Mizutani Y, Nakajima Y (2017). Impact of forward stroke volume response on clinical and structural outcomes after percutaneous mitral valve repair with MitraClip. Circ Cardiovasc Interv.

[23] Bhattacharyya S, Khattar R, Chahal N, Senior R (2014). Dynamic mitral regurgitation. Cardiol Rev.

[24] Lancellotti P, Gérard PL, Piérard LA (2005). Long-term outcome of patients with heart failure and dynamic functional mitral regurgitation. Eur Heart J.

[25] Barth S, Hautmann MB, Kerber S, Gietzen F, Reents W, Zacher M (2017). Left ventricular ejection fraction of < 20%: too bad for MitraClip©?. Catheter Cardiovasc Interv.

[26] Gasparini M, Muto C, Iacopino S, Zanon F, Dicandia C, Distefano G (2012). Low-dose dobutamine test associated with interventricular dyssynchrony: A useful tool to identify cardiac resynchronization therapy responders: Data from the LOw dose DObutamine stress-echo test in Cardiac Resynchronization Therapy (LODO-CRT) phase 2 study. Am Heart J.

[27] Da Costa A, Thévenin J, Roche F, Faure E, Roméyer-Bouchard C, Messier M (2006). Prospective validation of stress echocardiography as an identifier of cardiac resynchronization therapy responders. Hear Rhythm.

[28] Lee R, Haluska B, Leung DY, Case C, Mundy J, Marwick TH (2005). Functional and prognostic implications of left ventricular contractile reserve in patients with asymptomatic severe mitral regurgitation. Heart.

[29] Iacopino S, Gasparini M, Zanon F, Dicandia C, Distefano G, Curnis A (2010). Low-dose dobutamine stress echocardiography to assess left ventricular contractile reserve for cardiac resynchronization therapy: data from the Low-Dose Dobutamine Stress Echocardiography to Predict Cardiac Resynchronization Therapy Response (LODO-CRT) trial. Congest Heart Fail.

[30] Moss R, Bar S, Chandavimol M, Munt B, Thompson C, Abel J (2014). Contractile reserve induced with dobutamine echocardiography predicts outcome in patients with left ventricular dysfunction and mitral regurgitation. J Heart Valve Dis.

