# Supplementary figures and images for: Can stress echocardiography identify patients who will benefit from percutaneous mitral valve repair?
Source: Int J Cardiovasc Imaging. 2018 Nov 29;35(4):645–51. doi: 10.1007/s10554-018-1507-x (PMC6482124; doi:10.1007/s10554-018-1507-x)

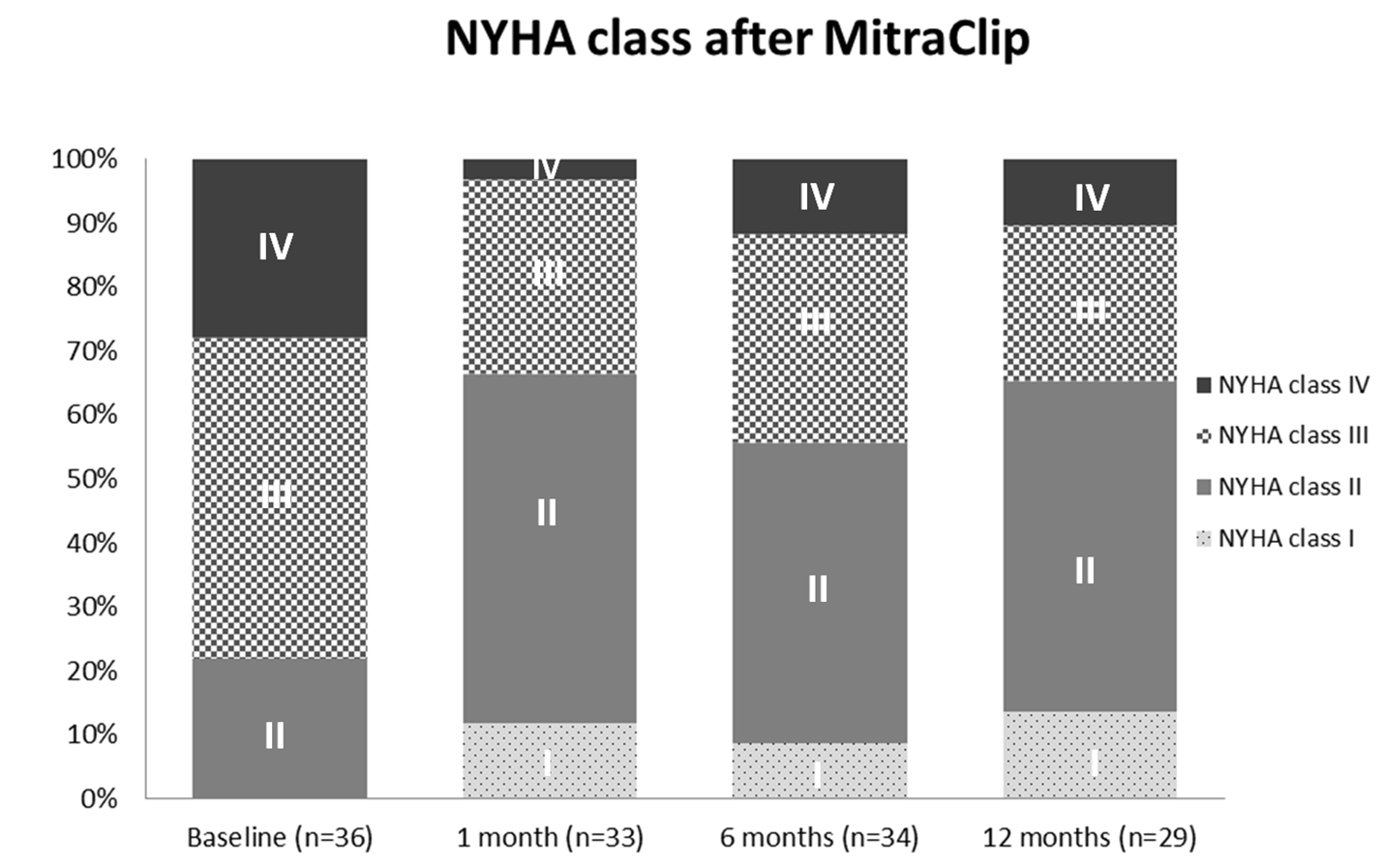

Supplement: Supplementary file 1 — Supplementary Fig. 1—NYHA: New York Heart Association (TIF 496 KB) [file 10554_2018_1507_MOESM1_ESM.tif]

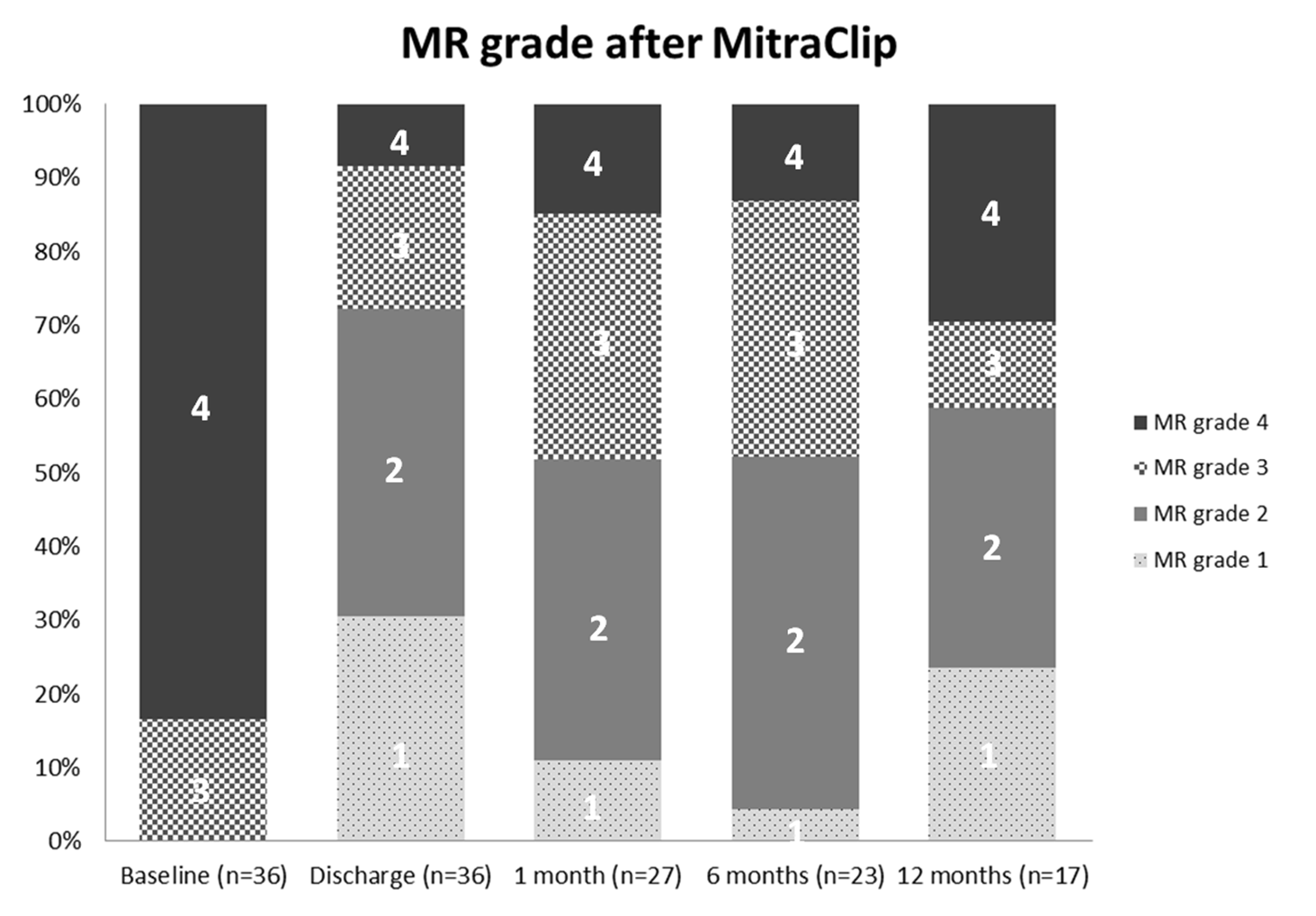

Supplement: Supplementary file 2 — Supplementary Fig. 2—MR: mitral regurgitation (TIF 526 KB) [file 10554_2018_1507_MOESM2_ESM.tif]
